# Association between the non-high-density lipoprotein cholesterol to high-density lipoprotein cholesterol ratio (NHHR) and angina pectoris in US adults: a cross-sectional retrospective study based on NHANES 2009–2018

**DOI:** 10.1186/s12944-024-02343-2

**Published:** 2024-10-26

**Authors:** Ying Cui, Mankyu Choi

**Affiliations:** 1 Department of Public Health Science, Graduate School and Transdisciplinary Major in Learning Health Systems, Graduate School, Korea University, Seoul, South Korea; 2 School of Health Policy & Management, College of Public Health Science and Transdisciplinary Major in Learning Health Systems, Graduate School, Korea University, Seoul, South Korea

**Keywords:** NHHR, Angina pectoris, NHANES, Restricted cubic spline model, Random forest analysis, Boruta algorithm

## Abstract

**Background:**

The non-high-density lipoprotein cholesterol to high-density lipoprotein cholesterol ratio (NHHR) plays a potential role in cardiovascular diseases. However, its association with angina pectoris remains unclear. Herein, we aimed to explore their relationship.

**Methods:**

This cross-sectional retrospective study included the 2009–2018 data from 22,562 adults diagnosed with angina pectoris, retrieved from the National Health and Nutrition Examination Survey (NHANES) database. NHHR was estimated from laboratory data, and angina pectoris diagnosis was ascertained from the NHANES questionnaire.

**Results:**

Angina pectoris risk was greater in the highest than in the lowest NHHR tertile (odds ratio [OR] = 1.61; 95% confidence interval (CI), 1.15–2.54; *P* = 0.006). Weighted logistic regression showed a positive association between NHHR and angina pectoris in the fully adjusted model (OR = 1.17; 95% CI, 1.07–1.28; *P* = 0.001). Restricted cubic spline analysis showed a linear association (*P* = 0.6572). Subgroup analyses indicated no significant differences across different stratifications (*P* > 0.05, all). Random forest analyses and Boruta algorithm corroborated that NHHR is a strong predictor of angina pectoris. Among the eight machine-learning models evaluated for predictive capabilities, the logistic regression model demonstrated the strongest predictive capability, with an area under the curve of 0.831.

**Conclusions:**

Our study suggests that NHHR is a risk factor for angina pectoris and may be used for risk prediction and to inform future intervention programs to reduce its incidence.

**Supplementary Information:**

The online version contains supplementary material available at 10.1186/s12944-024-02343-2.

## Background

Ischemic heart disease (IHD) is a leading cause of mortality and morbidity worldwide, contributing to a significant disease burden globally [1, 2]. One common clinical manifestation of IHD is angina pectoris, which is chest discomfort characterized by a constricting pain resulting from inadequate oxygen supply to the heart [3]. Its estimated prevalence ranges from 2% to over 10% in the general adult population and demonstrates an increasing trend over time [4–7]. Angina pectoris poses a significant threat to human life and health, leading to decreased quality of life and imposing a substantial economic burden on global healthcare systems [8, 9]. Accordingly, it is of utmost importance to identify modifiable risk factors of angina pectoris to guide effective prevention and interventions.

Angina pectoris results from a complex interplay of genetic, environmental, dietary, lifestyle, and metabolic factors [10]. Abundant evidence has shown that angina pectoris is associated with certain sociodemographic variables, such as sex, age, race, marital status, and education; lifestyle, such as smoking and alcohol use; and comorbidities, such as hypertension, diabetes, and cancer [5, 11]. Moreover, metabolic factors, such as lipid metabolism disorders, are particularly noteworthy as they can directly lead to coronary artery narrowing and myocardial ischemia by altering the progression of atherosclerosis, thus causing angina pectoris [12, 13]. Specifically, lipid-related profiles, such as low-density lipoprotein cholesterol (LDL-C) and high-density lipoprotein cholesterol (HDL-C), have recently been confirmed to be associated with the risk of angina pectoris, with increasing evidence consistently showing that increased LDL-C, non-HDL-C, and the LDL-C/HDL-C ratio are risk factors for angina pectoris [14–16].

Recently, a novel composite lipid indicator, the non-HDL-C-to-HDL-C ratio (NHHR), has gained recognition for its enhanced precision in predicting various diseases, including osteoporosis [17], kidney stones [18], breast cancer [19], diabetes [20], depression [21], periodontitis [22], and suicidal ideation [23]. The NHHR has the advantage of integrating both the risk (non-HDL-C) and protective (HDL-C) factors of angina pectoris, thus providing a more comprehensive risk prediction. However, large-scale studies exploring the association between the NHHR and angina pectoris are still lacking.

To fill this research gap, we conducted the current study to examine the association between the NHHR and angina pectoris based on the National Health and Nutrition Examination Survey (NHANES) database. We hypothesized that the NHHR is positively associated with the risk of angina pectoris. We utilized various analytic approaches, including weighted logistic regression, restricted cubic spline (RCS), subgroup analysis, random forest analysis, Boruta algorithm, and eight machine-learning models to confirm the link between them. Our findings would provide new insights into the underlying mechanisms of angina pectoris. Furthermore, understanding the role of NHHR in angina pectoris could be crucial for developing targeted prevention and treatment strategies, thereby contributing to improved care and management of this prevalent and burdensome condition.

## Methods

### Study design and participants

This study was reported according to the Strengthening the Reporting of Observational Studies in Epidemiology (STROBE) guidelines [24]. This study utilized data collected from the NHANES, which is a nationwide survey that uses a stratified, complex, multistage sampling methodology to assess the health and nutritional status of adults and children in the US [25]. The NHANES is completed by approximately 5,000 Americans annually and collects extensive health and nutrition-related data, including information regarding sociodemographic characteristics, dietary patterns, and health conditions. Data collection involves face-to-face interviews and comprehensive physical examinations, including physiological measurements and laboratory tests. All participants provided informed consent, and the survey methods, including the informed consent procedures, were approved by the National Center for Health Statistics Ethics Review Board. More detailed information is available online (https://www.cdc.gov/nchs/nhanes/index.htm). The current study did not require additional ethical approval or informed consent owing to its nature as a secondary data analysis.

We extracted data from the NHANES for the years 2009–2018 for a cross-sectional analysis. We selected eligible participants from an initial pool of 49,693 individuals based on the following inclusion and exclusion criteria: First, we excluded participants with missing data for the angina pectoris variable, including cases with “Don’t know” or “Refused” responses (21,221 individuals). Second, we excluded individuals with missing data for the NHHR variable (2,506 individuals). Lastly, we excluded participants with incomplete data on any covariates (3,404 individuals). This included missing information on the poverty-to-income ratio (2,473 individuals), body mass index (272 individuals), smoking (10 individuals), hypertension (32 individuals), diabetes (590 individuals), cancer (10 individuals), education level (13 individuals), and marital status (4 individuals). After these exclusions, a total of 22,562 participants were included in the final analysis.

### Assessment of the NHHR

The data source for the NHHR calculations was derived from laboratory data in the NHANES called ‘HDL.Doc’ for HDL-C data and ‘TCHOL.Doc’ for total cholesterol (TC) data. The NHHR was calculated based on the ratio of non-HDL-C (TC minus HDL-C) to HDL-C as follows: NHHR = (TC − HDL-C) / HDL-C [23]. All cholesterol measurements were expressed in milligrams per deciliter (mg/dL). Based on previous studies [20, 23], we divided participants into three groups according to NHHR tertiles: Tertile 1 (reference group), Tertile 2, and Tertile 3.

### Assessment of angina pectoris

Angina pectoris was ascertained by the NHANES questionnaire data file named “MCQ.Doc,” which assesses a participant’s history of angina pectoris by a self-reported question, ‘Have you ever been told that you had angina/angina pectoris’? This assessment approach has been validated by previous studies, confirming the accuracy of self-reported angina pectoris history [26]. Respondents who answered ‘yes’ were classified as having a history of angina pectoris. Additionally, the collected data represent the lifetime prevalence of angina pectoris rather than the incidence of new cases during the study period. Participants who did not provide any information regarding angina pectoris were excluded from the study.

### Covariates

To control for potential confounders, we included a range of covariates related to the NHHR and angina pectoris, including sociodemographic characteristics, lifestyle behaviors, and health indicators. The sociodemographic characteristics included age, sex, race, education level, marital status, and the PIR. For lifestyle behaviors, we assessed smoking behaviors by one question: ‘Have you smoked more than 100 cigarettes in your lifetime’? with optional answers being yes/no. Health status involved BMI (measured as a person’s weight in kilograms divided by the square of height in meters) directly obtained from the physical examination reports and a history of hypertension, diabetes, and cancer based on the self-reported questionnaire.

### Statistical analysis

All statistical analyses were performed using R (version 4.2; R Foundation for Statistical Computing, Vienna, Austria). The data were weighted according to the standard sample weighting guidelines of the NHANES to ensure sample representativeness. We first compared the sample characteristics between participants with and without angina pectoris using the chi-squared test for categorical variables and the Wilcoxon rank-sum test for continuous variables. We subsequently used multivariable logistic regression models to investigate the association between the NHHR (as a continuous variable) and angina pectoris using odds ratios (ORs) and 95% confidence intervals (CI). We further divided the NHHR into tertiles and compared the risk of angina pectoris among various tertile groups, with the lowest tertile serving as the reference.

We established three models to control for confounding factors and used weighted multivariable regression models to estimate the associations between NHHR and angina pectoris. Model 1 was not adjusted for any covariates. Model 2 was adjusted for age, sex, race, education level, marital status, and PIR. Model 3 (fully adjusted model) was further adjusted for BMI, smoking, hypertension, diabetes, total cholesterol, and cancer. Additionally, RCS regression was used to analyze the dose-response relationship between the NHHR and risk of angina pectoris. Moreover, we performed random forest analysis and used the Boruta algorithm to assess variable importance.

In addition, we created and evaluated eight machine-learning models using relevant variables from a fully adjusted model to predict angina pectoris. The models included logistic regression, Naive Bayes, random forest, boosted trees, support vector machine with radial basis function (RBF) kernel, multi-layer perceptron, K-nearest neighbors, and decision tree. We assessed their performance by plotting receiver operating characteristic (ROC) curves and calculating the area under the curve (AUC) values. Furthermore, we detailed the AUC, precision, accuracy, recall, and F1-Score of the top-performing model.

To assess the robustness of the findings across different sample characteristics, we conducted subgroup analyses based on sex, race, marital status, education level, smoking, hypertension, diabetes, and cancer. A two-sided *P*-value < 0.05 was considered statistically significant. Variance inflation factor (VIF) values were calculated to test collinearity, with VIF < 5 indicating no collinearity.

## Results

### Sample characteristics of the participants

Table 1 summarizes the demographic details of the participants. Of the 22,562 participants included in the final analysis, 574 reported a history of angina pectoris (2.54%). The participants’ mean age was 47.26 ± 16.87 years, 48.12% of them were male, and their mean NHHR was 2.88 ± 1.43 mg/dL. Notably, individuals with angina pectoris had a significantly higher NHHR than those without angina pectoris (3.06 ± 1.57 vs. 2.87 ± 1.42 mg/dL, *P* < 0.001). The two groups also showed significant differences in all other sample characteristics, including sex, age, race, marital status, education level, PIR, BMI, smoking status, hypertension, diabetes, and cancer (all *P* < 0.05).

**Table 1:** Sample characteristics and comparison between participants with and without angina pectoris (weighted)

Characteristic	Total, *N* = 193,876,28^a^	No angina pectoris, *N* = 189,760,203^b^	Angina pectoris, *N* =4,116,080^b^	*P*-value^c^
Sex				0.011
Male	10,898 (48.12%)	10,593 (47.95%)	305 (55.64%)	
Female	11,664 (51.88%)	11,422 (52.05%)	242 (44.36%)	
Race				< 0.001
Mexican American	3,193 (8.27%)	3,141 (8.37%)	52 (3.64%)	
Non-Hispanic Black	2,247 (5.74%)	2,196 (5.78%)	51 (3.81%)	
Non-Hispanic White	9,376 (67.26%)	9,057 (66.99%)	319 (79.38%)	
Other Hispanic	4,637 (10.48%)	4,552 (10.54%)	85 (7.54%)	
Other Race	3,109 (8.25%)	3,069 (8.31%)	40 (5.63%)	
Marital Status				< 0.001
Married	11,564 (55.35%)	11,280 (55.35%)	284 (55.13%)	
Widowed	1,678 (5.36%)	1,582 (5.26%)	96 (14.88%)	
Divorced	2,486 (10.33%)	2,389 (10.17%)	97 (17.66%)	
Separated	751 (2.36%)	737 (2.38%)	14 (1.45%)	
Never married	4,201 (18.28%)	4,171 (18.57%)	30 (4.90%)	
Living with partner	1,882 (8.22%)	1,856 (5.36%)	26 (5.97%)	
Education Level				< 0.001
Less than 9th grade	2,096 (4.80%)	2,023 (4.73%)	73 (7.75%)	
9–11th grade	2,905 (9.49%)	2,824 (9.44%)	81 (12.01%)	
High school graduate	5,071 (22.48%)	4,927 (22.36%)	144 (27.76%)	
Some college or AA degree	6,961 (32.03%)	6,800 (32.01%)	161 (32.98%)	
College, graduate or above	5,529 (31.21%)	5,441 (31.46%)	88 (19.50%)	
Smoking				< 0.001
Yes	9,839 (43.63%)	9,507 (43.21%)	332 (62.88%)	
No	12,723 (56.37%)	12,508 (56.79%)	215 (37.12%)	
Hypertension				< 0.001
Yes	7,996 (31.48%)	7,566 (30.47%)	430 (78.14%)	
No	14,566 (68.52%)	14,449 (69.53%)	117 (21.86%)	
Diabetes				< 0.001
Yes	3,047 (10.13%)	2,822 (9.48%)	225 (40.25%)	
No	19,515 (89.87%)	19,193 (90.52%)	322 (59.75%)	
Cancer				< 0.001
Yes	2,098 (10.14%)	1,980 (9.89%)	118 (22.02%)	
No	20,464 (89.86%)	20,035 (90.11%)	429 (77.98%)	
Age	47.26± (16.87)	46.91± (16.77)	63.49± (12.86)	< 0.001
PIR	2.99± (1.66)	3.00± (1.66)	2.59± (1.55)	< 0.001
BMI (kg/m^2^)	29.17± (6.92)	29.12± (6.91)	31.28± (7.19)	< 0.001
Total Cholesterol (mg/dL)	192.76± (41.59)	193.03± (41.48)	180.33± (44.66)	< 0.001
HDL-C (mg/dL)	53.81± (16.61)	53.92± (16.59)	48.43± (16.70)	< 0.001
NHHR (mg/dL)	2.88± (1.43)	2.87± (1.42)	3.06± (1.57)	< 0.001

^a^N not missing unweighted

^b^N (unweighted); (%) (weighted); Mean ± SD (weighted)

^c^chi-squared test with Rao & Scott’s second-order correction; Wilcoxon rank-sum test for complex survey samples

NHHR: Non-HDL-C to HDL-C ratio, HDL-C: High-density lipoprotein cholesterol, PIR: Poverty to Income Ratio, BMI: Body mass index, SD: standard deviation

### Association between NHHR and angina pectoris

Table 2 shows the association between the NHHR and angina pectoris using multiple logistic regression models, with and without controlling for covariates. Our results consistently showed a significant positive association between the NHHR and angina pectoris in the unadjusted model 1 (OR = 1.08, 95% CI: 1.00–1.16, *P* = 0.038), partially adjusted model 2 (OR = 1.09, 95% CI: 1.01–1.10, *P* = 0.035), and fully adjusted model 3 (OR = 1.17, 95% CI: 1.07–1.28, *P* = 0.001). The final model showed that, for each unit increase in the NHHR, the risk of angina pectoris increased by 17%. The VIF values for all covariates ranged from 1.07 to 1.40, indicating no collinearity.

We further categorized the NHHR into tertiles and compared the risk of angina pectoris among various tertile groups using multiple logistic models. The results showed a significantly higher risk of angina pectoris in the highest tertile group of the NHHR than that in the lowest tertile group in the unadjusted model 1 (OR = 1.34, 95% CI: 1.03–1.75, *P* = 0.031), partially adjusted model 2 (OR = 1.34, 95% CI: 1.02–1.77, *P* = 0.038), and fully adjusted model 3 (OR = 1.61, 95% CI: 1.15–2.54, *P* = 0.006).

**Table 2:** Association between the NHHR and angina pectoris by multiple logistic models (weighted)

Exposure	Model 1		Model 2		Model 3	
	OR (95% CI)	*P*-value	OR (95% CI)	*P*-value	OR (95% CI)	*P*-value
NHHR	1.08 (1.00,1.16)	0.038	1.09 (1.01,1.18)	0.035	1.17 (1.07,1.28)	0.001
NHHR Tertiles						
Tertile 1	Reference		Reference		Reference	
Tertile 2	1.26 (0.95,1.67)	0.110	1.20 (0.91,1.60)	0.200	1.28 (0.93,1.77)	0.125
Tertile 3	1.34 (1.03,1.75)	0.031	1.34 (1.02,1.77)	0.038	1.61 (1.15,2.24)	0.006
*P* for trend		0.031		0.038		0.006

Model 1: Unadjusted model

Model 2: Adjusted for age, sex, race, education level, marital status, and the poverty-to-income ratio

Model 3: Additionally adjusted for BMI, smoking, hypertension, diabetes, total cholesterol, and cancer

NHHR: Non-HDL-C to HDL-C ratio, OR: odds ratio, CI: confidence interval

### RCS analysis

**Fig. 1:**
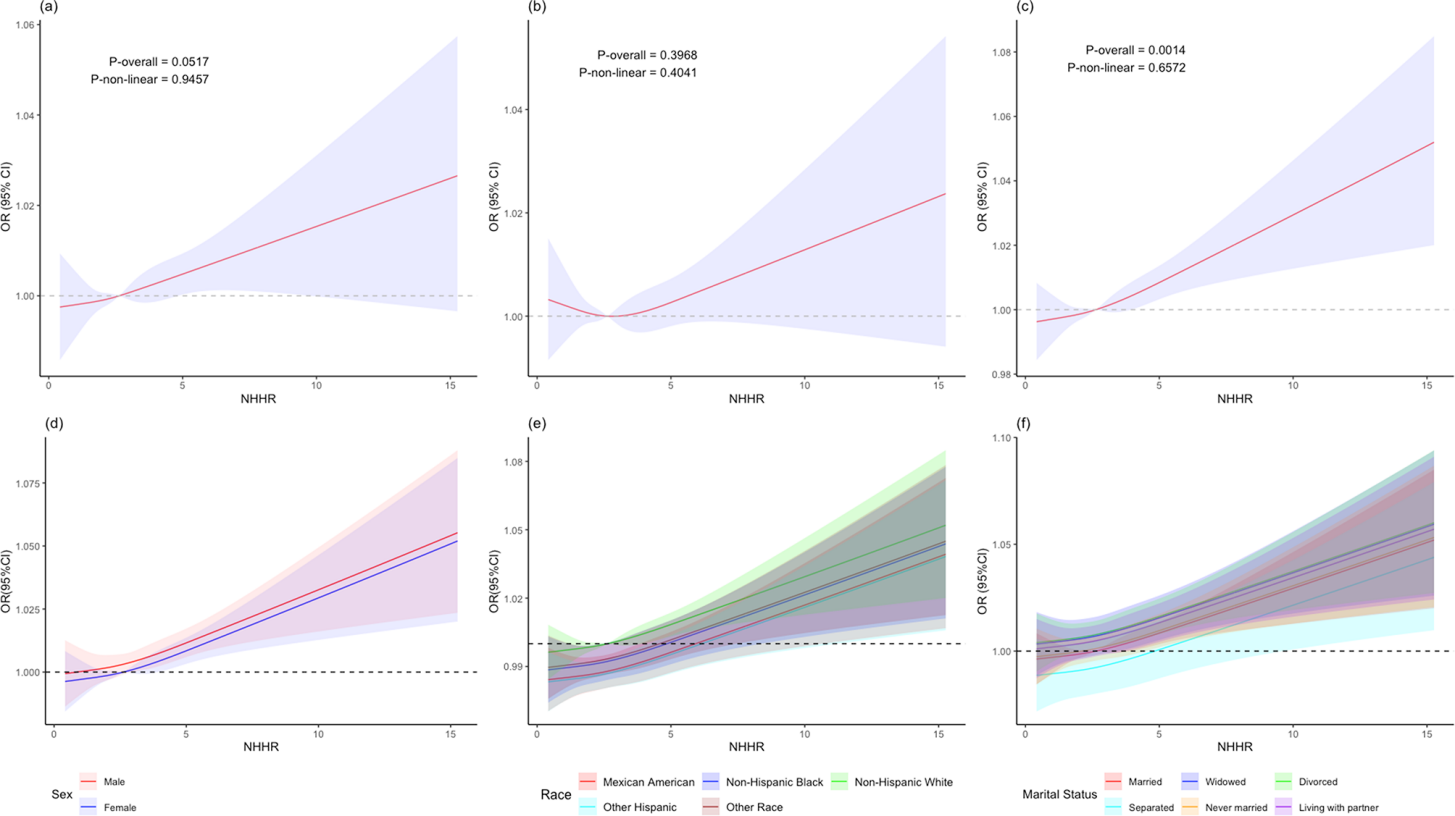
Restricted cubic spline curve for the association between NHHR and angina pectoris risk in model 1 (unadjusted) (**a**), in model 2 (adjusted for age, sex, race, education levels, marital status, and poverty-to-income ratio) (**b**), and in model 3 (adjusted for age, sex, race, education levels, marital status, poverty-to-income ratio, BMI, smoking, hypertension, diabetes, TC, and cancer) (**c**). Restricted cubic spline curve for the association between NHHR and angina pectoris risk in different sexes (**d**), in different races (**e**), and for different marital statuses (**f**). Lines represent odds ratios (OR), and colored areas represent 95% confidence intervals (CI). NHHR, non-high-density to high-density lipoprotein cholesterol ratio

Figure 1a–c shows the RCS curves to assess potential nonlinearity in the relationship between the NHHR and angina pectoris, with and without controlling for covariates. A positive linear relationship was observed between the NHHR and angina pectoris in the unadjusted model 1 (*P* for nonlinearity = 0.9457), partially adjusted model 2 (*P* for nonlinearity = 0.4041), and fully adjusted model 3 (*P* for nonlinearity = 0.6572), indicating a dose-response relationship. Furthermore, this positive association remained consistent across different characteristics such as sex, race, and marital status (Fig. 1d–f).

### Subgroup analyses

**Fig. 2:**
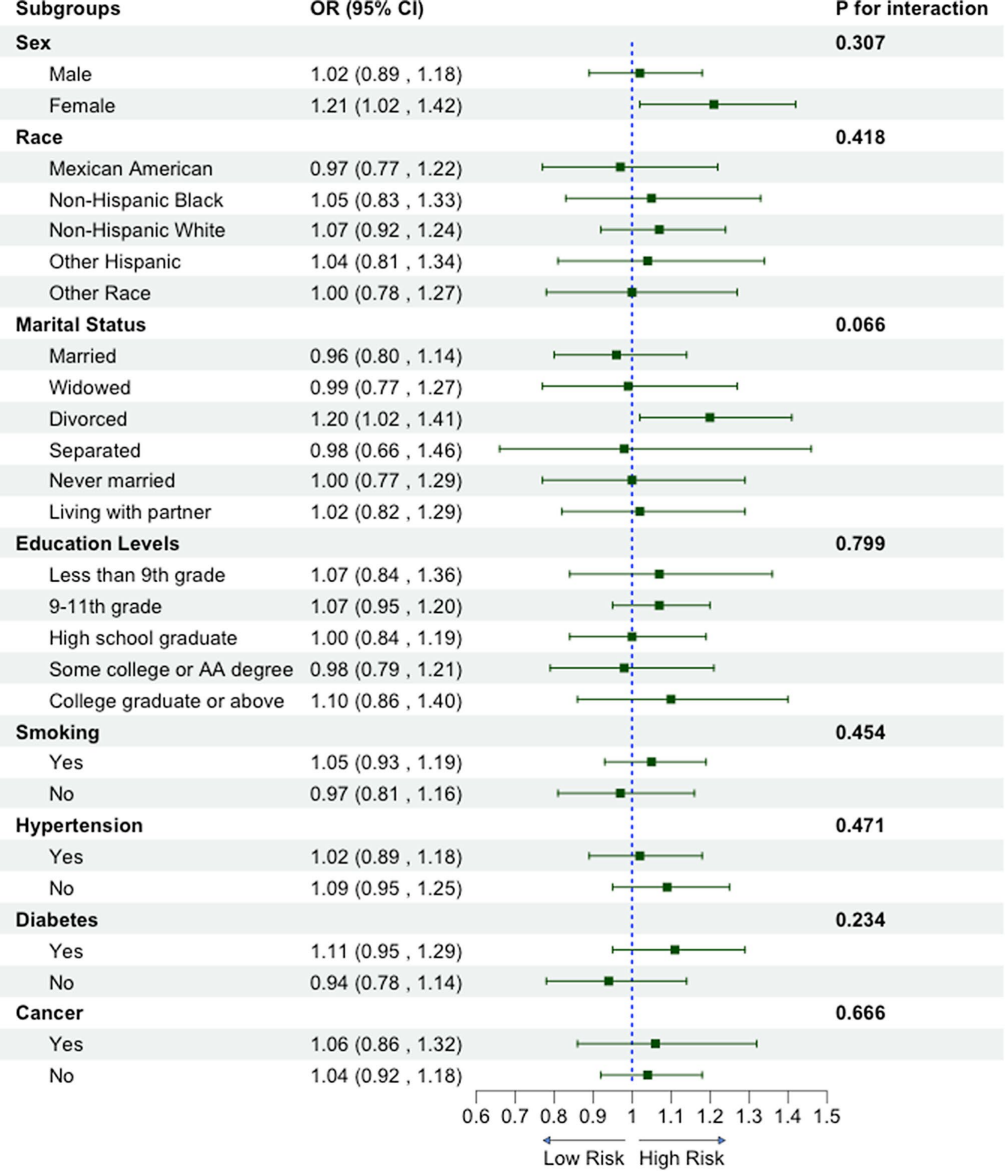
Subgroup analyses of the association between NHHR and angina pectoris. NHHR, non-high-density to high-density lipoprotein cholesterol ratio; OR, odds ratio; CI, confidence interval

Figure 2 presents the subgroup analyses of the association between the NHHR and angina pectoris based on sample characteristics, including sex, race, marital status, education level, smoking status, hypertension, diabetes, and cancer. The results showed a consistently significant association between the NHHR and angina pectoris after stratification, and no significant interaction was observed across the subgroups (*P*_interaction_ > 0.05 in all cases).

### Random forest analysis

**Fig. 3:**
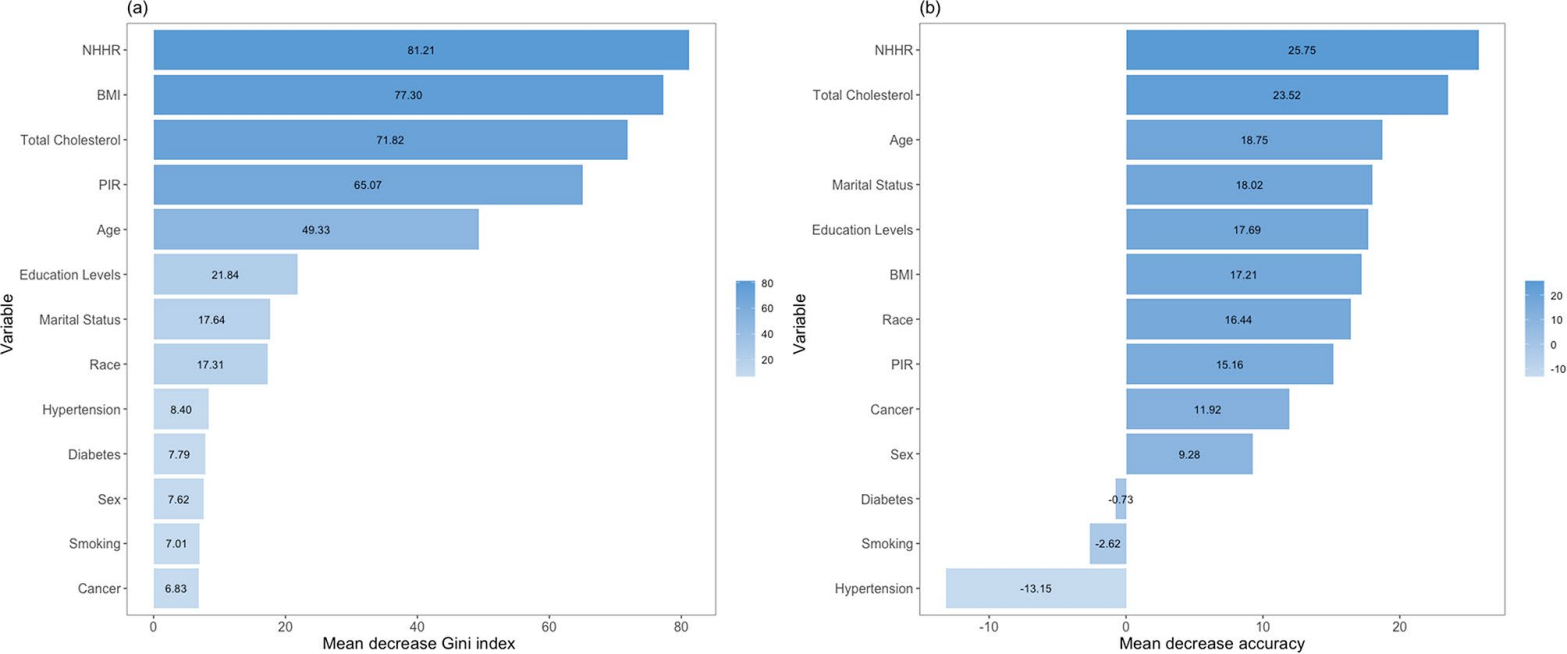
Variable importance according to the random forest analysis, evaluated by mean decrease in the Gini index (**a**) and mean decrease in accuracy (**b**). NHHR, non-high-density to high-density lipoprotein cholesterol ratio; BMI, body mass index; PIR, poverty-to-income ratio

Additionally, we conducted random forest analyses based on a fully adjusted model to identify the variables most closely associated with angina pectoris. Figure 3 presents the variable importance scores from the random forest analyses. The higher the values of the mean decrease in the Gini index and mean decrease in accuracy for a variable, the greater the importance of the variable in predicting angina. According to the mean decrease in the Gini index, the top three most important predictors were NHHR, BMI, and TC. When considering the mean decrease in accuracy, the top three predictors were NHHR, TC, and age. Evidently, NHHR ranked first in both the mean decrease in the Gini index and mean decrease in accuracy.

### Boruta algorithm

**Fig. 4:**
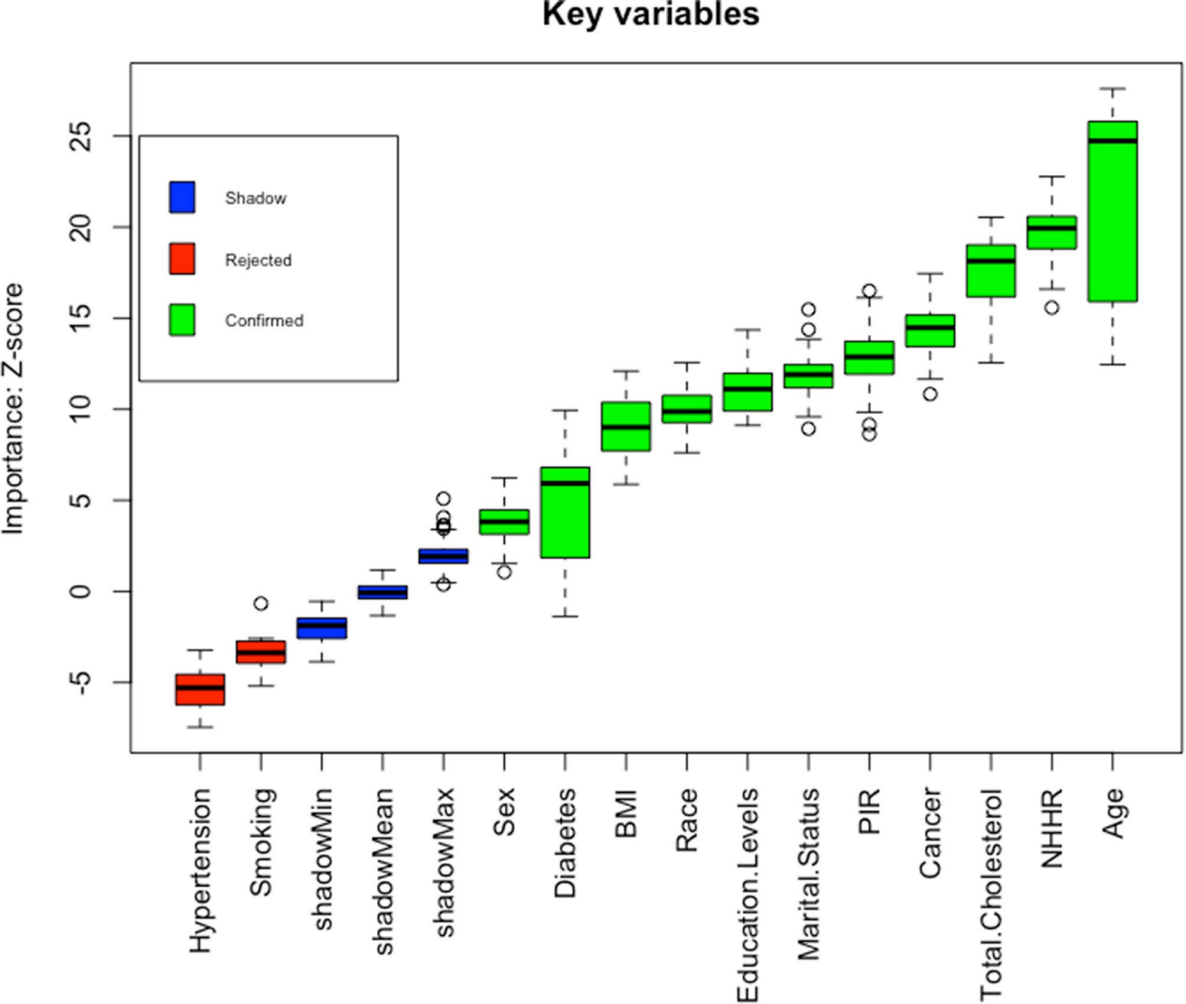
Predictor importance for angina pectoris according to the Boruta algorithm. A predictor was deemed important if its mean importance Z-score was significantly higher than the maximum value of the shadow variables (blue). Conversely, a predictor (red) was excluded if its mean importance Z-score was significantly lower than the maximum value of the shadow variables. NHHR, non-high-density to high-density lipoprotein cholesterol ratio; BMI, body mass index; PIR, poverty-to-income ratio

The feature selection results based on the Boruta algorithm are shown in Fig. 4. After 500 iterations, we identified 11 variables most closely related to angina pectoris. These variables, ranked by their z-scores, were age, NHHR, total cholesterol, cancer, PIR, marital status, education levels, race, BMI, diabetes, and sex. Thus, it can be concluded that the most important predictor is age, followed by NHHR.

Although smoking and hypertension were omitted because of their lower z-scores compared with those of the strongest related or shadow features, they were still included in subsequent analysis based on previous research and clinical experience.

### Performance of the eight machine-learning models

**Fig. 5:**
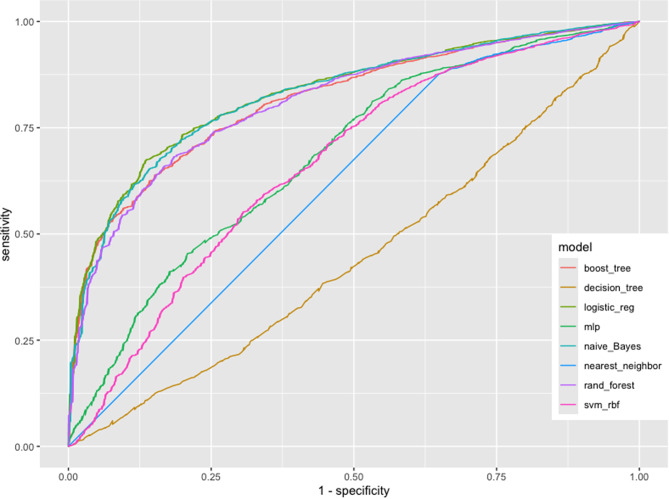
Receiver operating characteristic (ROC) curves of the eight machine-learning models

We evaluated the predictive capabilities of eight machine-learning models by plotting ROC curves and calculating AUC values. As shown in Fig. 5, the AUC values for the training sets of these models ranged from 0.500 to 0.831. The AUCs for each specific models were as follows: logistic regression model, 0.831; Naive Bayes model, 0.826; random forest model, 0.813; boosted trees model, 0.811; support vector machine with RBF kernel model, 0.625; multi-layer perceptron model, 0.607; k-nearest neighbors model, 0.575; and decision tree model, 0.500. The logistic regression model had the highest AUC value, with an accuracy of 97.4%, a precision of 100.00%, a recall of 97.4%, and an F1-Score of 98.7%. Additionally, the AUCs for the training and test sets were 0.829 and 0.845, respectively (Figure S1), indicating that the logistic regression model exhibits relatively strong predictive performance for angina pectoris, with no evident signs of overfitting. Further details regarding the training and test sets are provided in Table S1.

## Discussion

To the best of our knowledge, this is the first study revealing the association between the NHHR and angina pectoris using nationwide survey data. A significant positive association between the NHHR and angina pectoris was confirmed, which remained consistent across various models and analytical methods. RCS indicated a linear dose-response relationship between the NHHR and angina pectoris. Moreover, the association did not significantly differ across different subgroups. NHHR ranked first in variable importance score according to random forest analysis and second only to age according to the Boruta algorithm, highlighting its potential value for the diagnosis of angina pectoris.

The evaluation of the predictive capabilities of eight machine-learning models provided further insights into the significance of NHHR. Among these models, logistic regression showed the highest AUC value and demonstrated superior performance in predicting the occurrence of angina pectoris, with remarkable accuracy, precision, recall, and F1-Score metrics. Additionally, Naive Bayes, random forest, and boosted trees had AUC values above 80%, indicating although logistic regression achieved the highest AUC, the other three models also demonstrated comparable predictive capabilities. The clinical relevance of NHHR is underscored by its identification as a crucial variable through both the Boruta algorithm and random forest analyses, as well as its robust performance in the predictive models. Our findings suggest that the NHHR is a risk factor for angina pectoris and has potential clinical applications for its risk prediction, thereby useful to guide further prevention and intervention.

Angina pectoris is one of the most common symptoms of the cardiovascular system; nonetheless, few studies have investigated the relationship between the NHHR and angina pectoris. Our study has expanded the existing evidence on the association between dyslipidemia and cardiovascular diseases. For instance, a study in a West African population indicated that the NHHR was positively correlated with increased self-reported cardiovascular disease incidence and might serve as a biomarker for cardiovascular disease risk in this population [27]. Another study in a Turkish population showed that the NHHR was superior to traditional and non-traditional lipid markers in predicting the development of no-reflow in patients with ST-elevation myocardial infarction (STEMI) [28]. Another study reported that the NHHR had a stronger association with thrombolysis in myocardial infarction frame count and had a higher ability to detect coronary slow flow (CSF) than other non-traditional lipid profiles [29]. Our study adds further evidence by demonstrating a positive correlation between the NHHR and angina pectoris. Participants in the highest tertile group of NHHR had a higher risk of angina pectoris than those in the lowest tertile group. This relationship persisted and became more pronounced after full adjustment and remained robust in subgroup analyses. Additionally, NHHR ranked first in importance according to the random forest analysis and second only to age according to the Boruta algorithm, underscoring its diagnostic value for angina pectoris. Our findings highlight the critical role of the NHHR in the pathogenesis of angina pectoris and suggest that the NHHR may be used as an emerging marker for angina pectoris. Furthermore, the logistic regression model holds promise as a future tool to assist clinicians in making effective clinical decisions and improve early screening and diagnosis of patients affected by angina pectoris.

The NHHR reflects the balance between HDL-C and non-HDL-C, two lipoproteins with different functions [30]. An elevated NHHR may indicate a lipid metabolism imbalance [31]. The potential mechanisms underlying the predictive role of NHHR for future angina pectoris risk are unclear, although they may relate to the biochemical properties of HDL and non-HDL cholesterol. HDL-C is composed of the smallest and densest lipoprotein particles and is well known for its cardioprotective effects [32–34]. HDL-C inhibits atherosclerosis through multiple mechanisms, including the reverse transport of cholesterol, antioxidant, anti-inflammatory, anti-apoptotic, and antioxidant effects [35–38]. An elevated NHHR implies a relative decrease in HDL-C, weakening its atherosclerosis-inhibiting effects and thereby increasing the risk of atherosclerosis, which is a leading cause of angina pectoris [5, 39]. In contrast, an increase in non-HDL-C facilitates the deposition of lipoproteins on arterial walls, which is associated with atherosclerosis and leads to plaque accumulation [40, 41]. This can exacerbate the risk of angina pectoris by affecting vasodilation capacity and blood flow regulation.

Furthermore, our study has profound clinical implications in the risk assessment, prevention, and treatment of angina pectoris. The identification of NHHR as a potential risk factor for angina pectoris suggests that NHHR can be used as a reliable, inexpensive, and readily accessible biomarker for the risk stratification of angina pectoris in assessing cardiovascular disease risk. By employing the NHHR in the risk assessment of angina pectoris, this study will help clinicians identify patients who are at high risk of angina pectoris to guide early prevention and intervention in routine practice. Regular monitoring of lipid status can help detect high-risk populations and intervene early. For instance, physicians may offer advice on lifestyle changes or adjust the plans of treatment based on NHHR values for a given patient. For patients with an increased NHHR, clinicians may recommend controlling their diet (such as a low-fat and high-lipoprotein diet) and exercising to promote lipid metabolism. Furthermore, NHHR can be used as a primary therapeutic target in patients with angina pectoris and enable long-term follow-up of the treatment effects in clinical practice. While these findings indicate that NHHR has clinical utility, it is important to acknowledge that further research is needed to validate its application in clinical settings. Looking forward, we aim to conduct more in-depth research, including prospective cohort studies to confirm the predictive value of NHHR, as well as interventional trials to assess the impact of modifying NHHR on reducing the incidence of angina pectoris. Additionally, exploring the molecular mechanisms underlying the association between NHHR and angina pectoris will provide insights into potential therapeutic targets.

### Strengths and limitations

This study utilized representative data from a nationwide survey spanning 10 years in the US. We examined the associations between the NHHR and angina pectoris using multiple methods, including weighted logistic regression, RCS, Random Forest analysis, Boruta algorithm, eight machine-learning models, and subgroup analysis. Our findings revealed a potential dose-response relationship between the NHHR and angina pectoris. We first identified the NHHR as a risk factor for angina pectoris, and this marker may be used for future risk prediction.

However, the study had several limitations. First, the cross-sectional design precludes the establishment of a causal relationship between the NHHR and angina pectoris, as there is a possibility of reverse causality. Determining this relationship would require future longitudinal studies. Second, data collection was mainly based on self-reported questionnaires, which may introduce recall bias and affect the reliability of the results. Future studies should consider adding objective measurements to conduct a more accurate assessment. Third, our subgroup analyses were limited to specific sample characteristics and may not have covered other potential confounders. Future studies should include a more extensive subgroup analysis.

## Conclusions

This study confirms the NHHR as a risk factor for angina pectoris, indicating that it may be used as an effective biomarker to predict the risk of angina pectoris. Our findings provide new insights into the link between lipid metabolism disorders and angina pectoris, indicating the potential clinical utility of NHHR in risk assessment. However, due to the cross-sectional nature of this study and the reliance on self-reported data, the results should be interpreted with caution, as the observed association does not establish causation. To validate the clinical application of NHHR, further research is necessary. We recommend conducting prospective cohort studies and mechanistic investigations to assess the predictive value and reliability of NHHR at risk of angina pectoris, which could help guide effective prevention and treatment strategies.

## Electronic supplementary material

Below is the link to the electronic supplementary material.

**Supplementary Material 1:Table S1.** Baseline characteristics of the training and test sets.

**Supplementary Material 2:Fig. S1**. AUC values for training and test sets in the logistic regression model for angina pectoris.

## Data Availability

No datasets were generated or analysed during the current study.

## Abbreviations

AUC: Area under the curve
BMI: Body mass index
CI: Confidence interval
HDL-C: High-density lipoprotein cholesterol
IHD: Ischemic heart disease
LDL-C: Low-density lipoprotein cholesterol
NHANES: National Health and Nutrition Examination Survey
NHHR: Non-high-density lipoprotein cholesterol to high-density lipoprotein cholesterol ratio
OR: Odds ratio
PIR: Poverty-to-income ratio
RBF: Radial basis function
RCS: Restricted cubic spline
ROC: Receiver operating characteristic
TC: Total cholesterol
VIF: Variance inflation factor
