# Supplementary material for: Association between the non-high-density lipoprotein cholesterol to high-density lipoprotein cholesterol ratio (NHHR) and angina pectoris in US adults: a cross-sectional retrospective study based on NHANES 2009–2018
Source: Lipids Health Dis. 2024 Oct 26;23:347. doi: 10.1186/s12944-024-02343-2 (PMC11514896; doi:10.1186/s12944-024-02343-2)
Supplement: Supplementary file 1 — Supplementary Material 1:Table S1. Baseline characteristics of the training and test sets. [file 12944_2024_2343_MOESM1_ESM.docx]

**Table S1.** Baseline characteristics of the training and test sets

| **Characteristic** | **Overall,**  N = 22562 | **Training set,**  N = 15793 | **Test set,**  N = 6769 | ***P*-value** |
| --- | --- | --- | --- | --- |
| Angina pectoris (%) | | | | 0.678 |
| No | 22015 (97.6) | 15415 (97.6) | 6600 (97.5) |  |
| Yes | 547 (2.4) | 378 (2.4) | 169 (2.5) |  |
| Sex (%) | | | | 0.910 |
| Male | 10898 (48.3) | 7624 (48.3) | 3274 (48.4) |  |
| Female | 11664 (51.7) | 8169 (51.7) | 3495 (51.6) |  |
| Age (mean (SD)) | 49.03 (17.59) | 48.95 (17.49) | 49.21 (17.82) | 0.311 |
| Race (%) | | | | 0.366 |
| Mexican American | 3193 (14.2) | 2247 (14.2) | 946 (14.0) |  |
| Non-Hispanic Black | 2247 (10.0) | 1559 (9.9) | 688 (10.2) |  |
| Non-Hispanic White | 9376 (41.6) | 6532 (41.4) | 2844 (42.0) |  |
| Other Hispanic | 4637 (20.6) | 3297 (20.9) | 1340 (19.8) |  |
| Other Race | 3109 (13.8) | 2158 (13.7) | 951 (14.0) |  |
| Education.Levels (%) | | | | 0.266 |
| Less than 9th grade | 2096 (9.3) | 1473 (9.3) | 623 (9.2) |  |
| 9–11^th^ grade | 2905 (12.9) | 1984 (12.6) | 921 (13.6) |  |
| High school graduate | 5071 (22.5) | 3564 (22.6) | 1507 (22.3) |  |
| Some college or AA degree | 6961 (30.9) | 4869 (30.8) | 2092 (30.9) |  |
| College, graduate or above | 5529 (24.5) | 3903 (24.7) | 1626 (24.0) |  |
| Marital.Status (%) | | | | 0.676 |
| Married | 11564 (51.3) | 8109 (51.3) | 3455 (51.0) |  |
| Widowed | 1678 (7.4) | 1153 (7.3) | 525 (7.8) |  |
| Divorced | 2486 (11.0) | 1726 (10.9) | 760 (11.2) |  |
| Separated | 751 (3.3) | 532 (3.4) | 219 (3.2) |  |
| Never married | 4201 (18.6) | 2935 (18.6) | 1266 (18.7) |  |
| Living with partner | 1882 (8.3) | 1338 (8.5) | 544 (8.0) |  |
| PIR (mean (SD)) | 2.48 (1.63) | 2.50 (1.64) | 2.44 (1.61) | 0.006 |
| BMI (mean (SD)) | 29.28 (7.08) | 29.30 (7.07) | 29.22 (7.12) | 0.431 |
| Total.Cholesterol (mean (SD)) | 191.41 (41.71) | 191.63 (41.44) | 190.89 (42.35) | 0.217 |
| Smoking (%) | | | | 0.475 |
| Yes | 9839 (43.6) | 6912 (43.8) | 2927 (43.2) |  |
| No | 12723 (56.4) | 8881 (56.2) | 3842 (56.8) |  |
| Hypertension (%) | | | | 0.267 |
| Yes | 7996 (35.4) | 5560 (35.2) | 2436 (36.0) |  |
| No | 14566 (64.6) | 10233 (64.8) | 4333 (64.0) |  |
| Diabetes (%) | | | | 0.691 |
| Yes | 3047 (13.5) | 2123 (13.4) | 924 (13.7) |  |
| No | 19515 (86.5) | 13670 (86.6) | 5845 (86.3) |  |
| Cancer (%) | | | | 0.231 |
| Yes | 2098 (9.3) | 1493 (9.5) | 605 (8.9) |  |
| No | 20464 (90.7) | 14300 (90.5) | 6164 (91.1) |  |
